# Post-Anæsthetic Broncho-Pneumonia

**Published:** 1912-03-09

**Authors:** Henry J. Nash

**Affiliations:** (Lond.), Late coach Anatomy and Physiology Cambridge University. 22 Dorset Gardens, Brighton.


### The Editor's Letter Box.

### POST-ANÆSTHETIC BRONCHO-PNEUMONIA.

*To the Editor of* The Hospital.

Sir,—I should like to be permitted to pass a few remarks upon anæsthesia, as the article which has been appearing in your columns upon post-anæsthetic broncho-pneumonia has proved of great interest to me.

There can be no doubt that bronchitis or a bronchitic diathesis invariably predisposes to the above condition.

The Wassermann reaction, as is suggested, is undoubtedly a link in the chain of circumstantial evidence, tending, as it does, to prove the dissociation theory. When teaching physiology in Cambridge University I invariably pointed out that anæsthesia was most probably due to a destruction of cytoplasm in the ganglionic cells.—Faithfully yours, Henry J. Nash (Lond.),

Late coach Anatomy and Physiology,
Cambridge University.

22 Dorset Gardens,
Brighton.

